# The Epidemic: Preventive Measures

**Published:** 1892-01-30

**Authors:** 


### The Hospital

A Weekly Journal of

**Science, Medicine, Nursing, and Philanthropy.**

*For Hospitals, Asylums, Infirmaries, Dispensaries, Medical Practitioners, Students, Nurses, and the Charitable Public.*

Vol. XI.—No. 279.] [Jan. 30, 1892.

### The Epidemic: Preventive Measures.

The alarming spread of the influenza epidemic in all directions has induced the medical authorities at the Local Government Board to make such efforts as seem possible in the direction of prevention. It is, perhaps, a pity that Dr. Thorne Thorne did not anticipate rather than follow the somewhat energetic complaints against the tardiness of his department which have appeared in the daily press. Scientific men will readily understand and sympathise with the inaction of the Local Government Board. But there is a popular as well as a scientific aspect of the question. It may be true that the authorities at the Local Government Board have not much laboratory knowledge of influenza; nor, indeed, so much practical knowledge of a grosser kind as they would desire. But, however small be the amount of knowledge possessed by scientific experts, it is quite certain that that possessed by the general public is a great deal less. Besides, it is to be borne in mind that the general public, beholding the spread of a devastating epidemic, and looking in vain for any well-directed attempts to check it, are apt to fall into panic, and to suffer a great deal of unnecessary and injurious anxiety in consequence. There is always something reassuring to the multitude in the intelligent action of constituted authorities; and we are glad, therefore, on many grounds, that the Local Government Board has at last made a well-intentioned effort to check the progress of the plague.

The public of our day differs a good deal from the populace of three or four centuries ago; as much, in fact, as modern men of science differ from their specialist ancestors of the Tudor period. Our public, therefore, has to be approached by the authorities in a different way. We cannot now proceed by the mere method of stern compulsion and rule. Intelligent persons, such as the great mass of our fellow-countrymen are, demand reasons for our rules. This demand the officials at the Local Government Board have done well to recognise. They have taken the public into their confidence, and the result will doubtless be that their recommendations will be seriously and intelligently considered, and carried into effect in a loyal spirit.

The circular of Dr. Thorne Thorne emphasises two points particularly—First, that influenza is now considered to be not only infectious, but infectious at a very early stage, and that it may remain infectious for at least eight days; secondly, that if people will recognise and carry out certain easy requirements of health they may either altogether avoid the disease, or experience it in a comparatively mild and harmless form. It will be necessary for heads of families, and indeed for younger people, to try to understand what is really required of them in the way of precaution, and to submit to some inconvenience in carrying out the published directions. In order to do this some amount of real study should be given to the leading points of the circular issued by the Local Government Board.

The influenza, says the circular, is possessed of a singular ability to disperse itself over a population with great rapidity, owing to its brief incubation period. The meaning of this is that there are not several days or a week or two between the date of infection, and the date when the proper symptoms of the disease show themselves in their full development, as is the case with small-pox, scarlet fever, and some other well-known infectious diseases. On the contrary, there are hardly any prodromata at all, as the doctors call them; but a patient may be infected one day, and prostrate with the disease in a well-marked form almost within thirty-six or forty-eight hours. This rapidity of incubation gives practically no time for the isolation of the patient, and so we may have a whole household down in rapid succession within a day or two of each other. It is plain, therefore, that during an epidemic of such intensity as the present, every person who feels unusually chilly and depressed should take to his bed at once, and send for the doctor. For, although the illness may prove to be nothing more than an ordinary cold or a disordered stomach and liver, it is better to spend one or two days in bed for a comparatively trifling ailment than to run the risk of infecting the whole house with a severe and contagious disease. On the point of this rapidity of incubation and infection one statement made by Dr. Thorne Thorne is quite startling in the prospect of universal pestilence which it holds out. "The influenza," says this authority, "can give rise to some thousand attacks in the time that small-pox or typhus had taken to produce ten; each of the thousand cases being ready to infect other susceptible people, and the difficulty of applying principles of isolation and disinfection being in like measure enormously enhanced."

Dr. Thorne Thorne points out with minute care the persons and circumstances which require to be most watchfully considered. The young and the robust, he thinks, are not likely to come to very much harm, even though they should contract the disease. But the aged and the infirm are in a very different case; to them, as many of us know by the experience of our own families, influenza is often a swift messenger of death. One cannot help thinking that the aged and infirm would in many cases do well to isolate themselves, even by way of anticipating and preventing an attack. That is to say, they might keep their rooms and partly their beds, for a few days or weeks, and undergo a little prophylactic and strengthening medical treatment until the great wave of the epidemic has commenced to definitely subside.

In the case of schools and other institutions where large numbers of persons are gathered together, the advice given by the Local Government Board is to isolate any case at once, and with the utmost completeness. It is not because the disease is so dangerous to life that this measure is urged upon the authorities of institutions, but that it appears to be so rapidly and energetically contagious. A whole school, a whole office, a whole factory or workshop, may be down almost at once. Every medical man can confirm from his own practice, the observation that whole assemblages of persons are struck down as if simultaneously poisoned by one universally prevalent contagion. Schoolmasters are not generally too docile as pupils of others, much as they value docility in those whom they themselves teach. But they ought to take this small piece of rational instruction from recognised medical authority with intelligent candour, and to act upon it with all possible promptitude, alike in the interest of the general public, who may suffer by the concentration of the disease at particular centres, and of their own schools, whose activities may be brought to a peremptory standstill for the greater portion of a term, if the disease should once get beyond the power of control.

It has often been insisted upon in these pages that depressed conditions of the general health render all classes of persons peculiarly liable to attack. Exposure to cold and damp we have also held to be peculiarly dangerous. These views are emphatically confirmed by the circular of the Local Government Board. "The liability to contract influenza," says the circular, "and the danger of an attack if contracted, are increased by depressing conditions, such as exposure to cold or to fatigue, whether mental or physical. There is reason to believe that the development of an attack depends very largely on the receptivity of the individual; and that the power of resistance varies not only in different persons, but in the same person from time to time; being diminished by any conditions which depress the general bodily vigour. It is therefore important that at the time of an epidemic all persons should, as far as they are able, pay attention to such measures as tend to the maintenance of their health, wearing clothing of suitable warmth, and avoiding exposure to cold and fatigue, unwholesome food, and excessive use of alcoholic liquors."

It will be seen, as we said at the outset, that Dr. Thorne Thorne addresses himself to a rational people in a rational spirit. He takes it for granted that the demand for authoritative instruction put forward by the newspapers is a general demand, and that the instruction when given will be carried out with intelligence and thoroughness. People who expected the scientific heads of the Government Medical Department to furnish them with specifics for prevention, or nostrums for cure, will of course be disappointed with the circular of Dr. Thorne Thorne. But people of sense, who do not look for miracles when ordinary methods rationally carried out will serve their purpose, will be grateful for authoritative instructions of a definite kind, and will do their best to profit by them.

